# Age-specific Reference Intervals of Abbott Intact PTH—Potential Impacts on Clinical Care

**DOI:** 10.1210/jendso/bvae004

**Published:** 2024-01-12

**Authors:** Tejas Kalaria, Alexander J Lawson, Joanne Duffy, Ashishkumar Agravatt, Steve Harris, Clare Ford, Rousseau Gama, Craig Webster, Tarekegn Geberhiwot

**Affiliations:** Clinical Biochemistry, University Hospitals Birmingham NHS Foundation Trust, Birmingham, B15 2GW, UK; Clinical Biochemistry, University Hospitals Birmingham NHS Foundation Trust, Birmingham, B15 2GW, UK; Clinical Biochemistry, University Hospitals Birmingham NHS Foundation Trust, Birmingham, B15 2GW, UK; Biochemistry, PDU Medical College, Rajkot, 360001, India; Black Country Pathology Services, The Royal Wolverhampton NHS Trust, Wolverhampton, WV10 0QP, UK; Black Country Pathology Services, The Royal Wolverhampton NHS Trust, Wolverhampton, WV10 0QP, UK; Black Country Pathology Services, The Royal Wolverhampton NHS Trust, Wolverhampton, WV10 0QP, UK; School of Medicine and Clinical Practice, University of Wolverhampton, Wolverhampton, WV1 1LY, UK; Clinical Biochemistry, University Hospitals Birmingham NHS Foundation Trust, Birmingham, B15 2GW, UK; Centre for Endocrinology, Diabetes, and Metabolism, Queen Elizabeth Hospital, Birmingham, B15 2TH, UK; Institute of Metabolism and Systems Research, University of Birmingham, Birmingham, B15 2TT, UK

**Keywords:** age-specific PTH reference interval, parathyroid hormone, intact PTH, normocalcemic hyperparathyroidism

## Abstract

**Background:**

PTH assays are not standardized; therefore, method-specific PTH reference intervals are required for interpretation of results. PTH increases with age in adults but age-related reference intervals for the Abbott intact PTH (iPTH) assay are not available.

**Methods:**

Deidentified serum PTH results from September 2015 to November 2022 were retrieved from the laboratory information system of a laboratory serving a cosmopolitan population in central-west England for individuals aged 18 years and older if the estimated glomerular filtration rate was ≥60 mL/min, serum 25-hydroxyvitamin D was >50 nmol/L, and serum albumin-adjusted calcium and serum phosphate were within reference intervals. Age-specific reference intervals for Abbott iPTH were derived by an indirect method using the refineR algorithm.

**Results:**

PTH increased with age and correlated with age when controlled for 25-hydroxyvitamin D, estimated glomerular filtration rate, and adjusted calcium (*r* = 0.093, *P* < .001). The iPTH age-specific reference intervals for 4 age partitions of 18 to 45 years, 46 to 60 years, 61 to 80 years, and 81 to 95 years were 1.6 to 8.6 pmol/L, 1.8 to 9.5 pmol/L, 2.0 to 11.3 pmol/L, and 2.3 to 12.3 pmol/L, respectively. PTH was higher in women compared with men (*P* < .001). Sex-specific age-related reference intervals could not be derived because of the limited sample size.

**Conclusion:**

Age-specific Abbott iPTH reference intervals were derived. Application of age-specific reference intervals will impact the diagnosis and management of normocalcemic hyperparathyroidism, based on current definitions, and secondary hyperparathyroidism. Additional studies are required to clarify the effect of sex and ethnicity on PTH.

Hyperparathyroidism, characterized by excess secretion of PTH, is common and is classified as primary, secondary, or tertiary based on etiology [1]. Consistently elevated PTH levels with normal total and ionized calcium levels in the absence of a secondary cause of PTH elevation is termed normocalcemic primary hyperparathyroidism (NCPHPT) and is considered a variant of hypercalcemic primary hyperparathyroidism (PHPT) but its natural history is not fully understood [2, 3].

Despite ongoing efforts, commercially available PTH assays are not currently standardized [4]. Considerable method-related variation in PTH results limits the direct comparability of results from different clinical laboratories and research studies and hinders the development of universal reference intervals and action thresholds [4]. Method-specific reference intervals, often provided by the manufacturers, are used to interpret PTH results. It is widely accepted that assay differences are accounted for by method-specific reference intervals. However, method-specific reference intervals from historic studies used differing definitions of healthy individuals and may not prevent clinical management discordance in the diagnosis and management of parathyroid disorders [5, 6]. This is especially relevant to NCPHPT and secondary hyperparathyroidism, in which hypercalcemia is not a feature and the diagnosis is reliant on elevated PTH level [6].

An additional limitation of reference intervals from the literature is that most are not specific for age, sex, and ethnicity [5]. PTH concentration increases with age and the increase is independent of 25-hydroxyvitamin D, renal function, ionized calcium, and phosphate [7, 8]. The mechanisms for age-related PTH increase are not fully understood but may include age-related decrease in renal function, decreased synthesis, and increased degradation of calcitriol, decreased intestinal calcium absorption, and perhaps changes in calcium-sensing receptors in parathyroid glands [8-11]. A previous study reported a significant misclassification of PTH results when age-specific PTH reference intervals were compared with the manufacturer's single reference interval, especially in the elderly [12]. Age-specific PTH reference intervals for adults derived by indirect data mining methods for Roche and Siemens second-generation PTH (intact PTH [iPTH]) assays have been reported [12-14]. Age-specific reference intervals for children have been reported for several methods from the Canadian Laboratory Initiative on Pediatric Reference Intervals project [15-18]. However, age-specific reference intervals for adults are not available for the Abbott iPTH assay, which is one of the most widely used PTH assays by laboratories worldwide, including those in our region.

The gold standard for reference interval derivation is the direct method. The Clinical and Laboratory Standards Institute recommends at least 120 samples per group for the derivation of reference intervals [19]. This is difficult in practice if age-specific reference intervals are to be derived because this requires samples from a substantial number of healthy individuals depending on the number of age partitions. Additionally, the reference intervals derived by the direct method are subject to statistical uncertainty because of sampling bias. The sampling bias decreases with an increase in sample size, but for analytes with a skewed distribution like PTH, significantly more than 120 samples per age partition are required to minimize the sampling bias [20, 21]. This makes robust age-specific direct reference interval studies challenging and resource intensive. Health is relative without a universal definition, and an additional limitation of many direct method studies is the use of a strict definition of “health” when selecting reference individuals, and the studies are often influenced by cost and convenience factors rather than using population-level random selection. These selected “healthy” individuals may not represent the population tested in routine clinical practice [5, 22].

Indirect methods for the derivation of reference intervals achieve results comparable or superior to those by direct methods when the sample size is large and the fraction of pathological results in the dataset is relatively small (<20%) [21]. In addition to being derived from the subgroup of the population tested in the past, the reference intervals derived by indirect methods automatically factor in the preanalytical and analytical conditions and therefore better represent the “intent to test” scenario in practice for that population [22]. Of the computerized automatable indirect reference interval methods, the refineR algorithm achieves the lowest overall deviation from the true reference interval values in simulated datasets [21]. Furthermore, reference intervals derived from simulated datasets using the refineR algorithm were closer to the true reference intervals compared with the reference intervals derived by the direct method using 120 samples. For highly skewed simulated distributions, TSH for example, reference intervals derived using the refineR algorithm had less total error even when compared with the direct method with 400 samples [20].

The fraction of pathological results has a greater effect than sample size on the results of the indirect reference interval methods. Even with a relatively modest size of 1000 samples, the refineR method produces results comparable to the direct method with 120 samples for parameters with approximately normal distribution when the fraction of pathological samples was <30% and was not very inferior to the direct method for analytes with skewed and highly skewed distributions [21]. The performance of refineR and other indirect reference interval methods improve by minimizing the fraction of the pathological samples.

We therefore chose the refineR algorithm to derive age-specific Abbott iPTH reference intervals from the real-world data and to improve performance by minimizing the fraction of pathological results using stringent inclusion criteria at the point of data retrieval.

## Methods

Deidentified serum PTH results, estimated glomerular filtration rate (eGFR), 25-hydroxyvitamin D, and serum albumin-adjusted calcium and phosphate for outpatient samples received from September 2015 to November 2022 were retrieved for individuals aged 18 years and older from the laboratory information system of a Clinical Chemistry department serving a cosmopolitan population in the West Midlands region of England. Results were retrieved only if eGFR ≥60 mL/min, serum 25-hydroxyvitamin D > 50 nmol/L, serum albumin-adjusted calcium within the United Kingdom Pathology Harmony reference range of 2.2 to 2.6 mmol/L, and serum phosphate, where available, within the Pathology Harmony reference range of 0.8 to 1.5 mmol/L. Ionized calcium results were not available because it is not routinely measured for assessment of calcium status in outpatients. Serum albumin-adjusted calcium was used in preference to unadjusted total calcium for the selection of results because it is recommended by current parathyroid disorders guidelines [3, 23]. The serum 25-hydroxyvitamin D concentration cutoff was based on the United Kingdom National Institute for Health and Care Excellence sufficiency level, which is also the cutoff used by several other organizations worldwide [24, 25].

Serum PTH was measured on Abbott Architect i2000SR (Abbott Diagnostics, IL, USA). The Abbott PTH assay is a second-generation PTH assay and measures iPTH [4]. The long-term inter-assay coefficient of variation for iPTH was 8.0% at 3.7 pmol/L and 6.9% at 12.8 pmol/L. Serum albumin-adjusted calcium was reported by an in-house equation derived from a previously published method [26]. The equation was verified and updated every 2 years or earlier if indicated by bias in serum calcium or albumin assays. All the assays were ISO 15 189 accredited by the United Kingdom Accreditation Service.

Statistical analyses were performed using IBM SPSS Statistics for Windows, version 26 (IBM Corp.). Mann-Whitney *U* test and the Kruskal-Wallis H test were used to compare differences in 2 variables and 3 or more variables, respectively. Correlations were performed after the Box-Cox transformation of PTH.

Centile plots of PTH were prepared using GAMLSS (Generalized Additive Models for Location, Scale and Shape) version 5.4 in RStudio version 2022.07.2 + 576. *P*-splines using singular value decomposition function (pb()) was used in Box-Cox transformed distribution in the GAMLSS for the centile plots [27, 28]. The transformed distribution with smoothing decreases but does not abolish the effect of outliers and residual pathological results in the dataset on the centile plots. Therefore, the plotted centiles were not used as age-related reference intervals. Rather, the centile plots acted as a guide to the change in PTH with age and a rough guide to reference intervals with age. The plotted centiles were used to select the age partitions, in conjunction with the number of available results in each partition, for the age-specific iPTH reference intervals.

The reference intervals for the selected age bins were derived using the refineR algorithm version 1.5.1 in RStudio version 2022.07.2 + 576 [20]. The refineR algorithm derives reference intervals from a mixed distribution of pathological and nonpathological real-world test results. The algorithm assumes that most data in the set consist of nonpathological results that can be modeled with a Box-Cox transformed normal distribution, and the proportion of pathological results is insignificant in a region of the concentration range.

The refineR algorithm first identifies the main peak of the data distribution and computes a histogram around this main peak. The algorithm then uses regularized maximum likelihood optimization to estimate parameters of multiple iterations of Box-Cox transformed normal distributions. Though skewed, the distribution of PTH was not much shifted from zero and therefore 1-parameter Box-Cox transformation was applied [29]. The optimal transformed model is identified from the iterations by the algorithm based on the minimum cost function of goodness-of-fit in the original nontransformed PTH concentration domain. The cost function considers the likelihood of observed values in the histogram accounted for by the proposed model and the robustness of the model in the presence of pathological sample results. The nonpathological distribution in the identified optimal model is then used to derive reference interval percentiles [20, 29]. One thousand bootstrap iterations were performed for each age partition. The median of the 1000 bootstrap percentile estimates for the 2.5th, 50th, and 97.5th percentiles of the respective age partition was reported as point estimates along with 90% CIs. The 2.5th and 97.5th percentiles were reported as lower and upper reference intervals, respectively.

## Results

**Figure 1.**
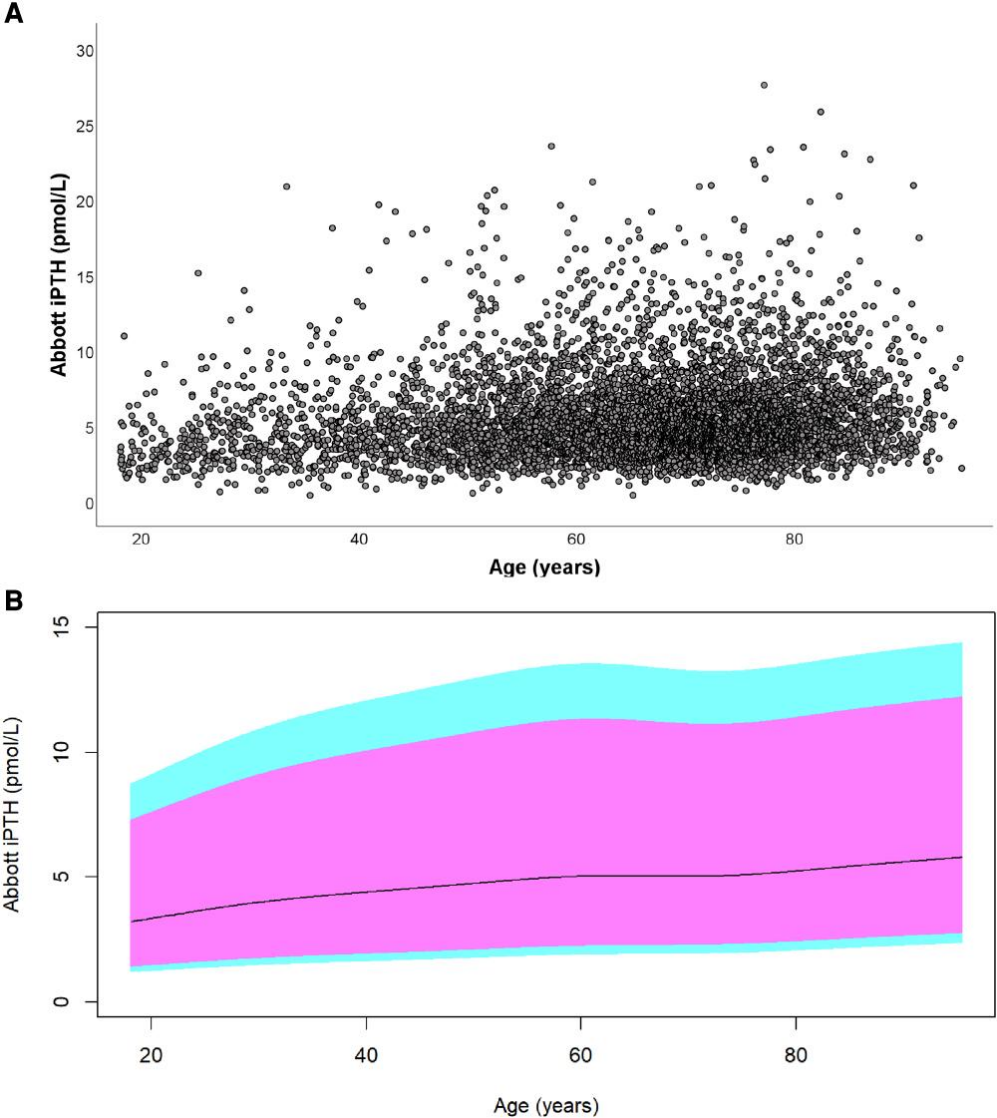
(A) Serum Abbott iPTH results and (B) percentiles of Abbott iPTH results with age (n = 5935). The 2.5th, 5th, 50th, 95th, and 97.5th percentiles are plotted in ascending order.

Of the 5937 sets of retrieved results fulfilling the inclusion criteria, 2 outlying sets with PTH of 50.0 pmol/L and 44.7 pmol/L in a 21-year-old male and 71-year-old female, respectively, were excluded. Of the remaining 5935 sets of results from individuals aged 18 to 95 years (median, 68 years; interquartile range [IQR]: 56-76), 82% were from women (n = 4867, median age 69 years; IQR: 57-76) and 18% were from men (n = 1068, median age 64 years; IQR: 51-75) (Fig. 1A). Median (IQR) eGFR, serum 25-hydroxyvitamin D, serum calcium, and serum albumin-adjusted calcium levels were 85 (74-90) mL/min/1.73 m^2^, 80 (66.1-96.5) nmol/L, 2.38 (2.30-2.45) mmol/L, and 2.37 (2.31-2.44) mmol/L, respectively. Serum phosphate was available for 515 sets and was 1.12 (1.00-1.23) mmol/L.

**Figure 2.**
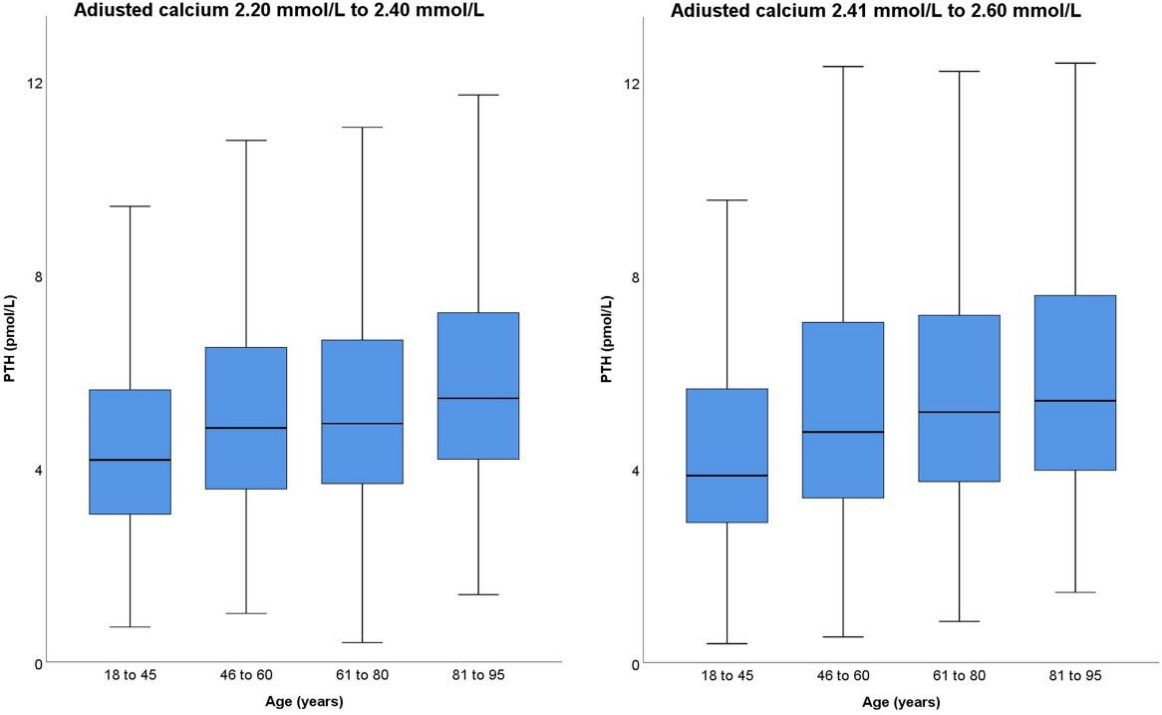
Age-related increase in PTH for adjusted calcium in lower and upper half of the reference interval (overall *P* < .001 and *P* < .05 for each age group and next age group).

**Figure 3.**
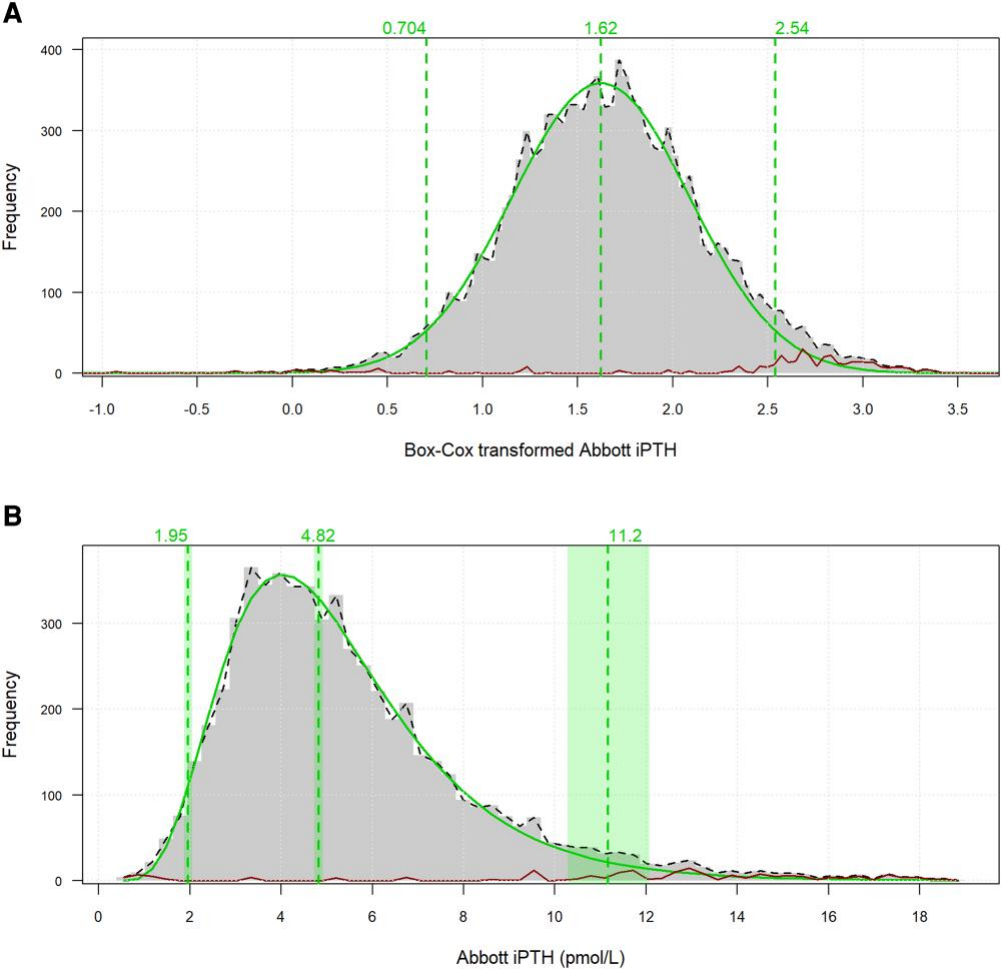
(A) Box-Cox transformed Abbott iPTH distribution using transformation factor λ = 0.0386 and (B) Abbott iPTH reference interval for age 18 to 95 years (complete dataset, n = 5935). In order of increasing PTH value, the vertical dotted lines indicate 2.5th, 50th, and 97.5th percentiles and the shaded regions encompassing the lines indicate 90% CIs of the percentiles. The optimal distribution of nonpathological results identified by the refineR algorithm is indicated by a solid green distribution. The red line closer to the abscissa indicates results that were not part of the identified optimal nonpathological distribution.

PTH correlated with age when controlled for 25-hydroxyvitamin D, eGFR, and albumin-adjusted calcium (*r* = 0.093, *P* < .001). The increase in PTH with age was consistent but a large proportion of PTH results were for ages between 60 and 80 years (Fig. 1A and 1B). Relatively fewer results for other ages limited the number of age partitions that could be created without undue widening of CIs of the derived percentiles. The PTH reference interval for the whole dataset and age-specific reference intervals for 4 age groups of 18 to 45 years, 46 to 60 years, 61 to 80 years, and 81 years and above were derived from at least 700 samples per age partition. PTH results in each selected age partition were lower than the next higher age partition (*P* < .05) and subsequent higher partitions (*P* < .001). The increase in PTH with age maintained for adjusted calcium in both lower and upper halves of the reference interval (Fig. 2). The refineR algorithm identified 95.3% of PTH results from the whole dataset and at least 91% of PTH results from individual age partitions as results constituting nonpathological distribution. The algorithm output for the whole dataset is plotted in Fig. 3 as an example. The lower reference limit (2.5th percentile), median, and upper reference limit (97.5th percentile) increased for each sequential age partition (Table 1). Both lower and upper reference limits for the 18- to 45-year group were 70% of the respective limits for the 80- to 95-year group.

**Table 1.** Serum Abbott iPTH reference intervals in adults

Group	Sample size	Lower reference limit*^a^* (pmol/L)	Median*^a^* (pmol/L)	Upper reference limit*^a^* (pmol/L)
All ages	5935	2.0 (1.9-2.1)	4.8 (4.7-4.9)	11.2 (10.3-12.1)
Aged 18-45 y	712	1.6 (1.2-1.8)	4.0 (3.7-4.2)	8.6 (6.9-10.7)
Aged 46-60 y	1259	1.8 (1.6-2.0)	4.6 (4.4-4.8)	9.5 (8.2-11.3)
Aged 61-80 y	3181	2.0 (1.9-2.1)	4.9 (4.7-5.0)	11.3 (9.9-12.0)
Aged 81-95 y	783	2.3 (1.6-2.4)	5.5 (5.0-5.6)	12.3 (9.1-13.4)
Female: all ages	4867	2.0 (1.9-2.1)	4.9 (4.7-5.0)	11.5 (10.4-12.5)
Male: all ages	1068	1.6 (1.0-1.8)	4.5 (4.2-4.7)	9.7 (8.0-11.3)

^*a*^The point estimates are the median of 1000 bootstrap iterations and numbers in the brackets indicate 90% CIs.

**Figure 4.**
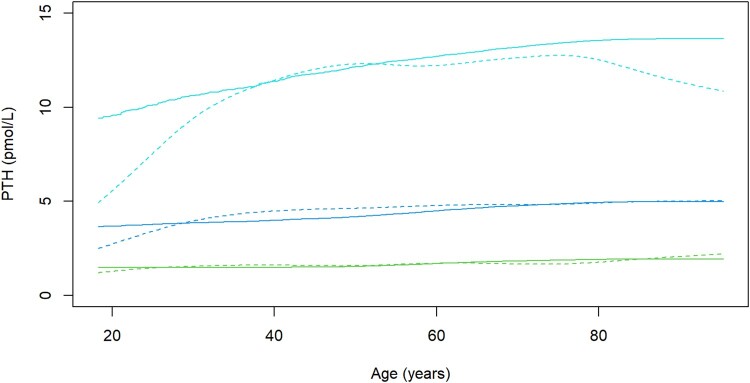
Comparison of PTH percentiles in women (solid lines, n = 4867) and men (discontinuous lines, n = 1068). The 2.5th, 50th, and 97.5th percentiles are plotted.

Serum PTH was higher in women (median, 5.0 pmol/L; IQR: 3.7-6.9) compared with men (median, 4.6 pmol/L; IQR: 3.3-6.2; *P* < .001). The difference was pronounced younger than age 30 years and older than age 85 years (Fig. 4); however, there were only 87 men younger than age 30 years and 40 men older than age 85 years in the dataset.

## Discussion

The age-related PTH increase independent of 25-hydroxyvitamin D and eGFR reported in this study is consistent with previous studies [8, 12, 30-33]. To our knowledge, this is the first study deriving age-related reference intervals for the Abbott iPTH assay in adults. Both the lower and upper reference limits of Abbott iPTH increased progressively with age and for the 18 to 45 years group both the lower and upper reference limits were 70% of the respective limits for the 80- to 95-year group. This is comparable to studies deriving age-specific PTH reference intervals in adults for other iPTH assays. The upper reference limit of PTH for the lowest of the age groups was 61%, 65%, and 68% of the upper reference limit for the highest of the age groups in indirect method reference interval studies for Siemens iPTH assay in an Australian population, Roche iPTH assay in a Spanish population, and Roche iPTH assay in a Brazilian population, respectively [12-14]. Although the change with age was similar, the absolute values of derived age-related reference intervals were different in all 3 studies, perhaps representing the differences in the iPTH assays and the need for assay-specific age-related reference intervals until the PTH assays are standardized [4, 6].

The use of a single method-specific reference interval in the diagnosis and management of parathyroid disorders has implications, in particular, for the diagnosis and management of NCPHPT and secondary hyperparathyroidism because hypercalcemia is not a feature and diagnosis depends on persistently elevated PTH. Moreover, the method-specific upper reference interval reported by many of the previous PTH reference interval studies is lower compared with that for Abbott iPTH derived in our study and Siemens iPTH in an Australian study. This difference may be due to underrepresentation of the elderly, as was often the case in older direct sampling reference interval studies [5, 12].

**Figure 5.**
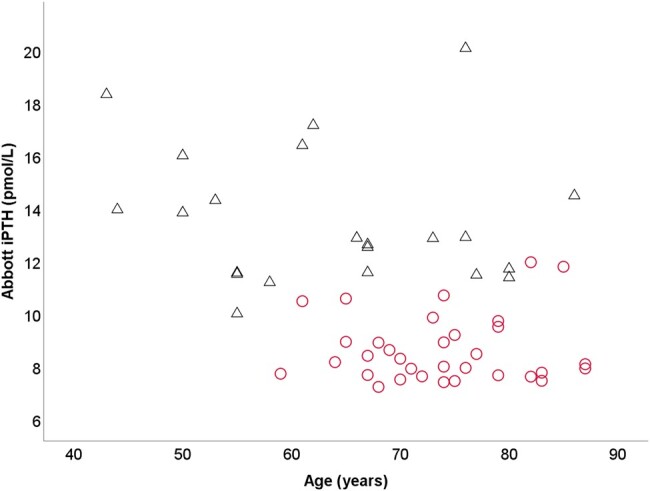
Change in the diagnosis of NCPHPT identified using manufacturer-provided Abbott iPTH reference interval when age-specific iPTH reference intervals derived in this study are applied. Of the 55 consecutively identified NCPHPT using the manufacturer-provided iPTH reference interval, PTH was within the age-specific reference interval in 33 patients (Ο) and above the age-specific reference intervals in 22 patients (Δ). Data were used with permission from Kalaria et al to generate transformative figure [6].

A previous study from the same laboratory assessing diagnostic discordance in NCPHPT using the manufacturer-provided single assay-specific reference intervals of Abbott, Roche, and Siemens iPTH assays identified 55 consecutive patients with NCPHPT in the Abbott assay laboratory. The manufacturer-provided Abbott iPTH reference interval of 1.6 to 7.2 pmol/L was used for case detection [6]. Of the 55 identified patients with NCPHPT in the laboratory with the Abbott method using the manufacturer-provided single method-specific reference interval, only 22 patients (40%) would have had NCPHPT if age-specific Abbott iPTH reference intervals derived in this study would have been applied (Fig. 5, data used with permission). Previous studies reporting on the prevalence and natural history of NCPHPT did not consider age-related PTH increase or use age-related reference intervals. The higher prevalence of NCPHPT reported in several studies, especially in the elderly, may be due to inappropriate overdiagnosis [34]. Of note was use of single PTH and albumin-adjusted calcium measurements and the nonavailability of ionized calcium results in this diagnostic discordance study comparing different laboratory methods. PTH has a circadian rhythm, the release is dynamic with pulses superimposed on basal secretion and it may be influenced by multiple factors including medications and calcium intake, which would not have been accounted for in this laboratory discordance study [2]. Generally, the recommendation for diagnosis of NCPHPT is to have normal albumin-adjusted total calcium and normal ionized calcium with elevated iPTH on at least 2 occasions over 3 to 6 months after secondary hyperparathyroidism has been excluded [2, 3].

The optimal PTH level in chronic kidney disease mineral bone disease is not known, in part because of differences in PTH results from different assays, and therefore the current Kidney Disease: Improving Global Outcomes recommendation is to base management decisions on trend from serial measurements [35]. It will be of interest if the expression of the PTH result as multiples of the age-related assays specific reference interval improve the utility of individual result in secondary hyperparathyroidism associated with chronic kidney disease.

PTH was higher in women than in men in this study. However, the sex difference in PTH concentration was mainly relevant under the age of 30 years and above the age of 80 years (Fig. 3). Varied sex differences in PTH and hyperparathyroidism are reported in the literature. A National Health and Nutrition Examination Surveys-based study found higher PTH levels in women compared with men, with ethnicity affecting the gender difference in PTH. The gender difference in PTH was highest in Blacks, followed by non-Hispanic whites, followed by Mexican-Americans [33]. Another epidemiological study in the United States reported Blacks had the highest incidence of PHPT followed by whites followed by Asians and Hispanics, and ethnic differences were seen in sex differences of the incidence [36]. In our study, the median PTH result in men was 8% lower than in women, which is similar to a laboratory database study from Western Australia in which men had approximately 8% lower PTH than women [8]. Other studies did not find a gender difference in PTH [12, 13, 37], although in 1 study a trend toward higher PTH values in women was observed in subjects with 25-hydroxyvitamin D > 50 nmol/L, but the difference did not reach statistical significance in any of the age groups studied [12]. Ethnicity may have influenced the sex difference observed in our study because the laboratory serves a cosmopolitan area where approximately one-third of the population is non-Caucasian, primarily of Asian descent but also Black and mixed ethnicities. Large sample size studies from a multiethnic population will be needed to clarify if, after adjusting for other affecting factors, sex has an effect on PTH concentration in some or all ethnicities. However, such a study will be difficult to perform by retrieving data from laboratory databases because ethnicity is usually not recorded. It is also possible that the sex difference observed in this study, especially the wider difference younger than age 30 years and older than age 80 years, was a statistical sampling error resulting from the PTH requesting practices in the region because there were very few PTH results in men outside the age bracket of 30 to 80 years.

The limitations of this indirect reference interval study are the absence of a very large sample size in each age group, a relatively small proportion of men in the study indicating the derived age-specific PTH reference intervals are particularly relevant for women, the inability to conclude a consistent sex effect on PTH, and the inability to derive sex-specific age-related PTH reference intervals because of sample size limitations. However, like all indirect reference interval studies, this study provides robust reference intervals for groups where PTH is measured more frequently in clinical practice. This is often not achievable in direct reference interval studies that generally enroll a comparable number of participants in each group, and the study size is often small because of resource limitation and practicality.

In addition to assay-related differences in age-related PTH reference intervals, there may be geographical differences in reference intervals because of differences in population (for example, ethnicity, age and sex distribution, deprivation, prevalence of negative calcium balance, proton pump inhibitor use, calcium and vitamin D supplementation practices), laboratory practices and method used to drive reference intervals as was found in a recent Abbott PTH reference interval study comparing 4 different UK sites [38]. PTH is more stable in EDTA plasma than in serum at room temperature [39]. Therefore, age-specific iPTH reference intervals may be slightly higher in EDTA plasma than in serum. However, many laboratories, including the laboratory in this study, preferentially use serum samples for PTH analysis for pragmatic reasons of minimizing the number of samples and blood volume in phlebotomy, and to enable reflective add-on of PTH by the laboratory to the existing serum sample if indicated by bone profile results. In addition to geographical and method-related differences, the use of serum for PTH analysis in the laboratory in this study could explain slightly low overall (age nonspecific) upper and lower reference intervals of PTH compared with another study in the UK population in which all 4 sites used EDTA plasma samples for PTH analysis [38]. Nonavailability of linked clinical data is a limitation of this and most other indirect reference interval studies. The combined prevalence of osteoporosis treatments, loop diuretics, and lithium treatment is unlikely to be high enough to have a marked effect on reference intervals derived by modern indirect reference interval methods [21]. However, in the absence of linked clinical data, we could not assess the effect of filtering for these factors. The use of albumin-adjusted calcium, and not ionized calcium, which is not routinely measured in community-dwelling individuals in most health care systems, may be considered another limitation of this and other indirect PTH reference interval studies.

In summary, this study confirmed an age-related increase in PTH and derived age-specific reference intervals for Abbott iPTH. Further studies are required to explore the effect of sex and ethnicity on PTH. Implementation of age-specific PTH reference intervals would decrease the inappropriate diagnosis of NCPHPT and associated additional testing and potentially avoidable parathyroidectomy in older subjects while enabling the diagnosis of NCPHPT where PTH is elevated compared with what is expected for the age in individuals fulfilling the rest of criteria.

## Data Availability

Some or all datasets generated during and/or analyzed during the current study are not publicly available but are available from the corresponding author on reasonable request.

## Abbreviations

eGFR: estimated glomerular filtration rate
iPTH: intact PTH
IQR: interquartile range
NCPHPT: normocalcemic primary hyperparathyroidism
